# Thermodynamics and Kinetics of Two-Dimensional H_2_ Gas on Ag(111) Studied by Tip-Enhanced Raman Spectroscopy

**DOI:** 10.1021/acs.nanolett.6c00325

**Published:** 2026-05-18

**Authors:** Shuyi Liu, Youngwook Park, Jun Yoshinobu, Takashi Kumagai, Martin Wolf, Akitoshi Shiotari

**Affiliations:** † Department of Physical Chemistry, Fritz-Haber Institute of the Max-Planck Society, Faradayweg 4-6, 14195 Berlin, Germany; ‡ Wuhan National Laboratory for Optoelectronics, 12443 Huazhong University of Science and Technology, Wuhan 430074, China; ¶ The Institute for Solid State Physics, The University of Tokyo, 5-1-5 Kashiwanoha, Kashiwa, Chiba 277-8581, Japan; § 88301 Institute for Molecular Science, National Institutes of Natural Sciences, Okazaki 444-8585, Japan; ∥ The Graduate University for Advanced Studies, SOKENDAI, Hayama 240-0193, Japan

**Keywords:** molecular hydrogen, surface diffusion, desorption, two-dimensional gas, tip-enhanced Raman scattering

## Abstract

Surface diffusion is an essential elementary process of molecules on surfaces which undergo low-dimensional transport, aggregation, and catalytic reactions; however, evaluating the thermodynamics and kinetics of adsorbates rapidly diffusing on a surface as a two-dimensional gas (2DG) remains challenging even for direct observation by scanning tunneling microscopy. In this study, we analyze thermodynamic and kinetic processes of H_2_ physisorbed on Ag(111) using tip-enhanced Raman spectroscopy (TERS) at 7–14 K. The temperature evolution of the TERS intensity of the H_2_ rotational transition reveals that the concentration of the H_2_ 2DG on the surface is maximum at 10–11 K, decreasing at lower temperatures due to molecular aggregation and as well at higher temperatures due to desorption from the surface. Based on Van’t Hoff and Arrhenius equations, the potential energy diagram of the molecular aggregation and desorption are evaluated, demonstrating the utility of TERS for analyzing surface dynamics.

## Graphical abstract

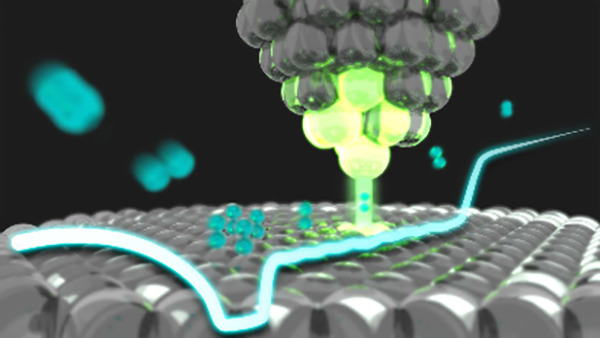

The diffusion of atoms and molecules adsorbed on solid surfaces plays an integral role in the chemical and physical phenomena at surfaces, such as crystal growth,^1,2^ atomic/molecular transport,^3,4^ molecular self-assembly,^5^ and heterogeneous catalysis.^6,7^ In addition to pioneering research using photoemission electron microscopy and field ion microscopy,^6,7^ the investigation of surface diffusion dynamics has been advanced using scanning tunneling microscopy (STM).^8^ Tracking a single adsorbate with an STM tip allows quantitatively evaluating thermodynamic processes, such as diffusion,^9–14^ desorption,^15^ and other motions.^16,17^ When adsorbed molecules experience low diffusion barriers thermally surmountable even at low temperatures, they form a two-dimensional gas (2DG)^18,19^ and are sometimes detected by STM as noise in the tunneling current.^8,19–24^ STM has also been used to characterize thermal equilibrium of the 2DG phase and condensed phases of the adsorbate, referred to as a two-dimensional solid (2DS), clarifying the delicate balance between molecule–surface and molecule–molecule interactions.^19,21,25^ However, when molecular diffusion occurs faster than the STM time resolution (typically ms), the sample is imaged as an apparently clean surface even in the presence of a 2DG. This has been reported for physisorbed molecules, such as hydrogen molecules on coinage-metal surfaces,^22,25,26^ making molecular detection challenging. Although several STM-based spectroscopic methods, such as differential conductance measurements, have characterized the vibrational properties and configurational switching of the metal–hydrogen–metal junctions,^16,25–32^ there is still a lack of microscopic insights on the thermodynamics and kinetics of a 2DG of weakly bound molecules.

Low-temperature tip-enhanced Raman spectroscopy (LT-TERS) based on STM can obtain local chemical information on single molecules adsorbed on surfaces.^33–35^ The STM junction under laser illumination acts as a plasmonic cavity, where a strongly localized electromagnetic field is generated by the surface plasmon resonance. The LT environment stabilizes the tip-apex structure, thereby confining an electromagnetic field at the subnanometer scale.^36^ While typically immobile molecules strongly adsorbed on surfaces have been investigated, LT-TERS has also great potential to precisely evaluate mobile molecules, such as molecular switches and diffusing molecules.^37–41^ Very recently, TERS of hydrogen molecules adsorbed on Ag(111) at 10 K has been reported;^42^ the rotational and vibrational Raman signals of hydrogen were detected from a Ag–Ag junction, although the molecules physisorbed on the surface have an ultralow diffusion barrier (*E*_diff_ ≲ 1 meV),^43^ making the 2DG invisible in STM images.^31^ Here we evaluate the diffusion, desorption, and 2DG–2DS phase transition of hydrogen molecules on Ag(111) by temperature-dependent TERS measurements.

For LT-TERS measurements, we position a Ag tip illuminated with a 532 nm laser above a clean Ag(111) surface at 10 K with the STM feedback loop closed. The STM unit was then exposed to H_2_ gas until H_2_-derived peaks appeared in TER spectra (∼30 Langmuir; see Methods). Under these conditions, a submonolayer coverage (less than 1 monolayer) of H_2_ on the surface is expected according to previous theoretical calculations.^42^ The topmost curve in Figure 1b shows a TER spectrum recorded after the gas exposure stopped. The broad peak at ∼150 cm^–1^ is assigned to Ag phonon modes of the tip,^42,44^ and its constant appearance over the temperature-dependent measurements assures no change in the tip structure. The 351 cm^–1^ peak is assigned to a rotational transition of H_2_,^42^ as the Raman-active transition between rotational quantum states from 0 to 2 has an energy of 354 cm^–1^ in the gas phase.^45^

**Figure 1:**
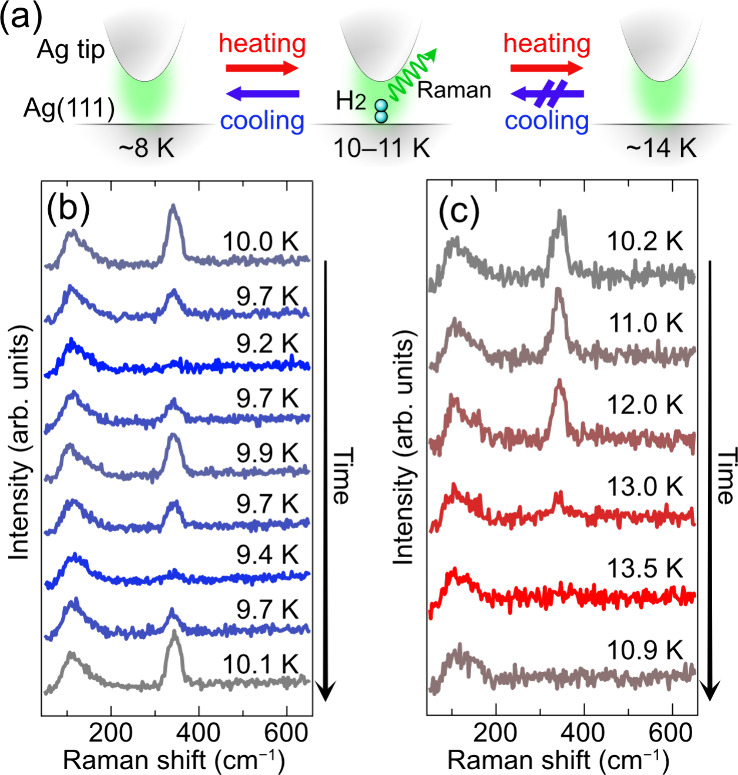
(a) Side-view schematic of the junction between a Ag tip and H_2_/Ag­(111) at different temperatures. The green halo represents localized surface plasmon resonance, providing enhanced Raman scattering from the junction. The Raman signal of H_2_ has a maximum intensity at 10–11 K, whereas it was lost below ∼8 K and above ∼14 K. (b) Time evolution of TERS for H_2_/Ag­(111) during repeated cooling-and-heating cycles between 9 and 10 K. (c) Time evolution of TERS for H_2_/Ag­(111) during a heating-and-cooling cycle above 10 K.

We investigated the temperature *T* dependence of the peak intensity *I* of the H_2_ rotational transition, as schematically summarized in Figure 1a. Figure 1b shows a series of TER spectra recorded during cooling-and-heating cycles. As *T* decreases from 10.0 K, *I* attenuates (Figure 1b, the second curve from the top) and eventually becomes smaller than the noise floor at 9.2 K (the third). After that, we repeated the heating processes to 10 K and cooling processes to 9 K and observed the peak reappearance and disappearance, respectively, multiple times (Figure 1b, from top to bottom). This suggests that the total number of H_2_ molecules on the surface was unchanged and that no molecule exists within the junction at *T* ≲ 9 K (Figure 1a, left). In the heating-and-cooling cycles with a sufficiently slow ramping rate (total duration of ∼1 h for the record in Figure 1b), *I* was uniquely determined for each *T* and no hysteresis was observed.

We also conducted TERS measurements at temperatures above 10 K. Figure 1c shows spectra obtained during a monotonic increase in *T* from 10.2 to 13.5 K (top four curves) and a spectrum recorded after cooling to 10.9 K (bottommost curve). *I* maximizes at 10–11 K but attenuates at higher *T* (see also Figure S1 in the Supporting Information (SI) for the time trace of the peak intensity). Notably, once the peak disappeared at 13.5 K, no molecular peaks were recorded at any temperatures. This indicates that H_2_ disappeared irreversibly from the junction (Figure 1a, right), unlike the reappearance during heating from 9 to 10 K (Figure 1b).

As described above, a characteristic *T* dependence of *I* was observed (see Figure S2 for reproducible measurements using different Ag tips). The irreversible decay of *I* at *T* ≳ 11 K (Figure 1c) can be attributed to thermal desorption of the molecules from the surface. In contrast, the attenuation of *I* at *T* ≲ 11 K (Figure 1b) seems counterintuitive because the diffusion rate is expected to decrease as *T* decreases. We attribute the attenuation of *I* observed in the low-*T* range to a decrease in the concentration of the H_2_ 2DG, as schematically shown in Figures 2a–c. When TERS detects diffusing H_2_ molecules, the Raman peak intensity is proportional to the residence time of molecules within the STM junction during the spectrum acquisition time (10 s). The decrease in the number of diffusing H_2_ molecules by cooling from 10 to 9 K can be explained by growth of local condensed islands on the surface, i.e., 2DG-to-2DS phase transition (Figures 2b to 2a). Conversely, heating from 9 to 10 K induces two-dimensional (2D) sublimation, i.e., 2DS-to-2DG phase transition, increasing the number of diffusing molecules (Figures 2a to 2b). At *T* > 11 K, thermal desorption decreases the 2DG concentration (Figures 2b to 2c), leading to the peak disappearance eventually.

**Figure 2:**
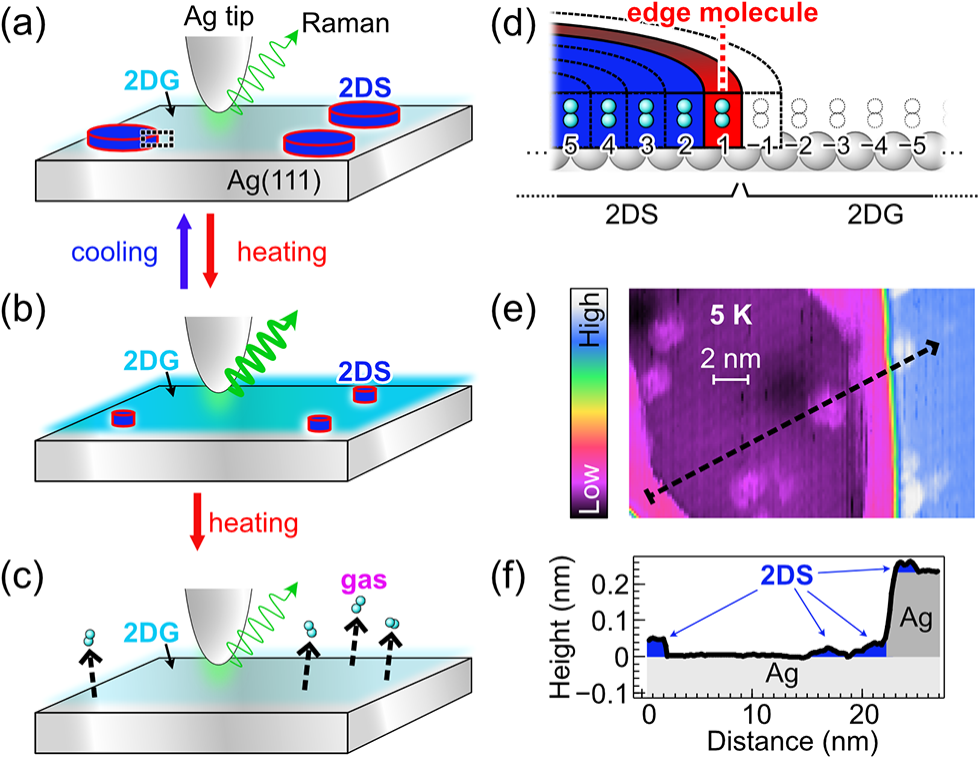
(a–c) Bird’s-eye-view schemes of a H_2_ 2DG on Ag(111) depending on *T*. The color intensity of the cyan halo depicts the concentration of the 2DG. The blue disks represent H_2_ 2DS islands. The thickness of the wave arrow from the STM junction illustrates the intensity of Raman scattering from the H_2_ 2DG. (d) Cross-section view of a 2DS–2DG boundary at the atomic scale, corresponding to the magnified area in the dotted black rectangle in (a). The site index (positive: 2DS, negative: 2DG) is labeled at the bottom of each adsorption site. The concentric disks depict the distributions of Sites −1 to 5. (e) STM image of H_2_ 2DS on Ag(111) at 5 K acquired using a different STM apparatus from the LT-TERS system. (f) Line profile along the black arrow in (e). Areas shaded in blue, light-gray, and dark-gray represent the H_2_ 2DS, lower Ag terrace, and higher Ag terrace, respectively.

Although the H_2_ 2DS is expected to nucleate at defects or atomic steps of Ag(111), such assemblies were observed by our LT-TERS system neither with STM imaging nor with TERS below 9 K even when the tip location was moved over the surface. This is plausible because the current noise of the LT-TERS apparatus used is too high to visualize the 2DS and/or small 2DS assemblies may easily diffuse during tip scanning. To verify the presence of the H_2_ 2DS on Ag(111), we used a different STM apparatus (see Methods). Figure 2e shows a typical STM image acquired by the apparatus at 5 K. Circular islands with a diameter of ∼2 nm on the terrace and thin bands attached near the Ag step edges are attributed to the 2DS (see also Figure S3 for other images by the STM apparatus). The island appearance supports the 2DS–2DG transition scheme in Figures 2a–c. As shown in the line profile (Figure 2f), the 2DS structures are much thinner (0.02–0.04 nm) than the single Ag-atom step (0.24 nm), implying the observation difficulty (see Figures S4 and S5 for STM images by the LT-TERS apparatus).

Thermodynamic and kinetic analysis can reveal the potential energy diagram for the described phase transitions. First, we focus on the low-*T* range, where the desorption from the surface is negligible (Figure 1b; see also Figure S1, showing the constant peak intensity for 1000 s at 10–11 K), and thus surface diffusion and the 2DG–2DS transition dominate the dynamics of H_2_. We note that, to minimize the possible influence of the tip-induced diffusion and desorption on the analysis, we used a relatively large tip–sample gap (tunneling current of 1 nA and sample bias voltage of 10 mV) for the TERS measurements. This mitigates the effect of local heating due to the plasmon decay on the *T*-dependence measurements. Although a previous study observed local heating at narrow Ag–Ag junctions by anti-Stokes scattering spectroscopy,^46^ no feature was detected under the conditions used in this study (see Figure S6 for the anti-Stokes scattering spectrum). Moreover, the use of the low bias suppresses tip-induced molecular motions due to the electric field or inelastic tunneling current.^23,32^ We thus conclude that the observed temperature dependence is dominated by the change in concentration of the H_2_ 2DG outside the STM junction (Figures 2a–c).

The 2DG concentration is determined by the size of the 2DS islands, as a decrease and increase in the island size correspond to the 2D sublimation (2DS-to-2DG transition) and the reverse process (2DG-to-2DS transition), respectively (Figures 2a and 2b). To describe the rate of the island growth, we use an established model of “2D islands + 2D gas” for crystal growth and thin-film formation.^47–49^ When molecules located at island edges (‘edge molecule’ on Site 1 in Figure 2d) diffuse to adjacent empty adsorption sites (Site −1) with a rate constant of *k*_1_, the island size is reduced. Conversely, when the edge molecules are located on Site 1, diffusion of 2DG molecules to Site −1 with a rate constant of *k*_–1_ results in the island growth. Within this model, the growth rate of the 2DS islands is thus governed by

1 $$\frac{dN_{2DS}}{dt}=-k_{1}\left(1-\theta_{2DG}\right)N_{1}+k_{-1}\theta_{2DG}N_{-1}$$

θ_2DG_ is the 2DG concentration that is defined by the number of diffusing H_2_ molecules relative to the number of adsorption sites outside the 2DS islands. *N*_2DS_ denotes the number density of the 2DS molecules over the surface, i.e., the number of molecules forming the 2DS islands per unit area. *N*_1_ and *N*_–1_ denote the number density of the edge molecules and the number density of empty adsorption sites directly adjacent to the edge molecules, respectively (see the concentric disks for Sites 1 and −1 in Figure 2d). When the 2D sublimation and desublimation reach equilibrium, i.e., d*N*_2DS_/d*t* = 0, the equilibrium constant of *K*_1_ ≡ *k*_1_/*k*_–1_ can be expressed as

2 $$K_{1}=\frac{N_{-1}}{N_{1}}\frac{\theta_{2DG}}{1-\theta_{2DG}}$$

The ratio *N*_–1_/*N*_1_ is a constant independent of the shape and number of the islands (see SI for details).

For the thermal equilibrium between the 2DG and 2DS phases at each *T*, the Van’t Hoff equation is applicable:

$$\frac{d}{dT}\ln K_{1}\left(T\right)=\frac{{\Delta H}_{sub}^{2D}}{k_{B}T^{2}}$$ 3

where *k*_B_ is the Boltzmann constant and ${\Delta H}_{sub}^{2D}$ is the enthalpy of the 2D sublimation.^18^ As shown by the potential energy diagram in Figure 3f, ${\Delta H}_{sub}^{2D}$ corresponds to the energy of a 2DG molecule relative to the energy of a molecule located at a 2DS island edge (i.e., ‘edge molecule’ in Figures 2d and 3e).

**Figure 3:**
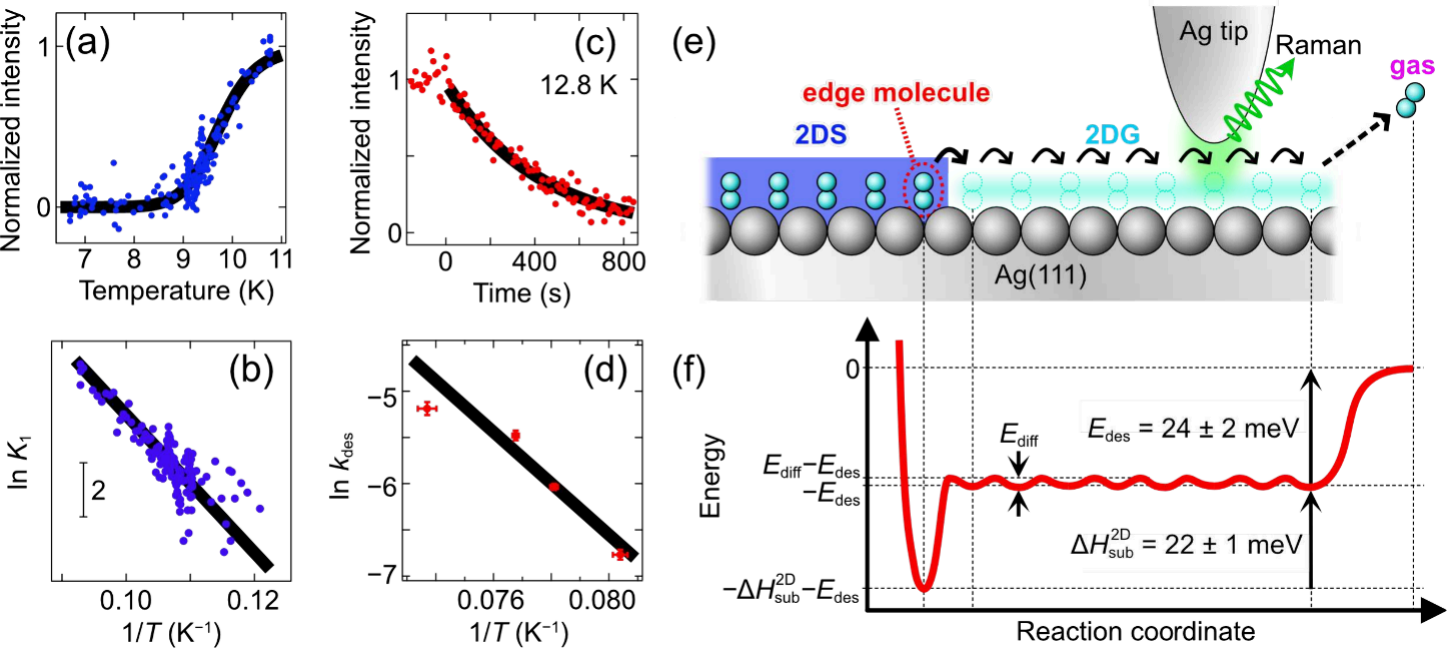
(a) Temperature *T* dependence of the peak intensity *I* at 351 cm^–1^. The black curve represent the *I*(*T*) curve reproduced from the fitting result shown in (b). (b) Van’t Hoff plot using the experimental *I*(*T*) plot. The black line indicates the fitting result by eq 4 with the parameter of ${\Delta H}_{sub}^{2D}=22\pm 1$ meV. (c) Time trace of the peak intensity at 351 cm^–1^ at a constant temperature of 12.8 K. The origin of the horizontal axis is the time when the gas exposure was stopped. The black curve represents the fitting result using eq 5. (d) Arrhenius plot using experimental *I*(*t*) plots. The measurements were conducted with the same tip as in (c). The horizontal and vertical error bars represent the standard deviations derived from *T* distribution during the *I*(*t*) measurement and from the curve fitting by eq 5, respectively. The black line indicates the fitting result by eq 6 with the parameters of *E*_des_ = 24 ± 2 meV and *A* = 10^6.8±0.7^. (e) Side-view schematic and (f) potential energy diagram for a H_2_ molecule located at a 2DS island edge (‘edge molecule’) to diffuse over an Ag terrace (‘2DG’) and to desorb from the surface (‘gas’). The energies in (f) are referenced to the gas-phase molecule (0 meV).

Using the Van’t Hoff plot, we evaluate the TERS peak intensity *I* as a function of *T*. Figure 3a shows a *I*(*T*) plot recorded while heating. No peak is observed below 9 K (i.e., *I* = 0), and the intensity maximizes at 11 K (*I* = *I*_max_), in good agreement with the data shown in Figure 1b recorded with a different tip in a narrower *T* range. As mentioned above (Figure 2a–c), the continuously varying intensity (0 ≤ *I* ≤ *I*_max_) resembles the concentration of the H_2_ 2DG, i.e., *I* = θ_2DG_ *I*_max_, under equilibrium conditions. From eq 2, the Van’t Hoff equation can be expressed as

$$\begin{array}{lll} \ln\frac{I\left(T\right)}{I_{max}-I\left(T\right)} & = & \ln K_{1}\left(T\right)+C_{1}\\ & = & -\frac{{\Delta H}_{sub}^{2D}}{k_{B}}\cdot \frac{1}{T}+C_{0} \end{array}$$ 4

where *C*_1_ ≡ ln­( *N*_1_ */*N**_–1_) and *C*_0_ are constants independent of *T* (see SI for details). Figure 3b shows the Van’t Hoff plot derived from the experimental *I*(*T*) plot in Figure 3a. A slope of ${\Delta H}_{sub}^{2D}=22\pm 1$ meV is obtained from the line fitting (black line in Figure 3b; see also the reproduced *I*(*T*) curve in Figure 3a).

Next, we analyze the data in the high-*T* range (Figure 1c). The Van’t Hoff plot described above (Figure 3b) predicts *k*_1_ to exceed 100*k*_–1_ at *T* ≳ 11 K, suggesting that the 2DS islands are spontaneously disassembled. Therefore, in the high-*T* range, we only consider desorption of H_2_ in the 2DG from the surface (Figure 2c) with a desorption rate constant of *k*_des_.

To analyze the irreversible peak disappearance above 12 K, we measured time (*t*) dependence of the TERS peak intensity at constant temperatures. Figure 3c shows a *I*(*t*) plot recorded at 12.8 K. For the measurements, we first exposed a clean surface at the set-point temperature to H_2_ gas until the peak intensity was saturated. After stopping the gas exposure (*t* = 0), we observed a monotonic decay of *I* corresponding to the decrease in the 2DG concentration θ_2DG_ (Figure 3c). Under the first-order desorption of H_2_ from Ag(111),^50^ the peak intensity is described as

5 $$I\left(t,T\right)=I_{max}\exp\left[-k_{des}\left(T\right)t\right]$$

The solid curve in Figure 3c shows the fitting result of the *I*(*t*) plot. The time-dependent measurements at several constant temperatures provide a series of *k*_des_(*T*) for an Arrhenius plot,

6 $$\ln k_{des}\left(T\right)=-\frac{E_{des}}{k_{B}}\cdot \frac{1}{T}+\ln A$$

where *E*_des_ represents the desorption energy and *A* is the pre-exponential factor. From the plot (Figure 3d), we obtain *E*_des_ = 24 ± 2 meV.

Finally, by analysis of the two separate *T* ranges (Figures 3b and 3d), we can derive the potential energy diagram of the phase transition and desorption for H_2_ on Ag(111), as shown in Figure 3f. The obtained desorption energy *E*_des_ = 24 ± 2 meV for the 2DG is in a good agreement with the previously reported value of 26 ± 2 meV determined by temperature-programmed desorption experiments.^50,51^ In contrast, previous theoretical calculations^42^ show a desorption energy of 87 meV/molecule for an adsorption structure of 0.69-monolayer H_2_ on Ag(111). Such a high-density structure should be analogous to the 2DS, where attractive intermolecular interactions stabilize the system. From the energy diagram (Figure 3f), the desorption energy for a molecule located at a 2DS island edge (‘edge molecule’) is calculated to be $E_{des}+{\Delta H}_{sub}^{2D}=46\pm 3$ meV. This value is in reasonable agreement with the previous calculations (87 meV), considering an edge molecule interacts with lower numbers of neighboring molecules than to those inside the island.

We note that in the above-mentioned reaction scheme (2DS ⇄ 2DG → gas), the tip–adsorbate interaction and its effect on the diffusion process are ignored (Figure 3e). Although the attractive interaction between the Ag tip and H_2_ is expected to be faint with the STM set point parameters used,^42^ the tip perturbation should be considered for STM-based thermodynamics and kinetic analyses in general. For completeness, we show in the SI an analysis using a more complicated reaction scheme that takes also into account molecular trapping at the STM junction site (Figures S7 and S8). As a result, the stabilization energy of a diffusing molecule in the STM junction is estimated to be only ∼1 meV, validating the original model (Figure 3f).

In summary, we demonstrate the thermodynamic and kinetic analysis of diffusion, desorption, and phase transition between 2DG and 2DS for hydrogen molecules on an Ag(111) surface using LT-TERS. The concentration of the 2DG determines the H_2_-derived peak intensity of TERS, which provides insights into the energetics of the thermal equilibrium between the 2DG and 2DS phases and the irreversible desorption process. As a result, the enthalpy of the 2DS-to-2DG sublimation is estimated to be 22 ± 1 meV and a desorption energy from the 2DG of 24 ± 2 meV. Such TERS-based quantitative analysis is potentially adaptable to various physisorption and weak chemisorption systems; TERS measurements of STM-observable 2DG–2DS equilibrium systems^21,22^ are highly promising to provide complementary insights on the phase transitions.

## Methods

The STM-based LT-TERS experiments were performed in an ultrahigh-vacuum (UHV) chamber (modified UNISOKU USM-1400). The single-crystal Ag(111) surface (MaTeck) was cleaned by Ar-sputtering-and-annealing cleaning cycles in the chamber. A resistance cryogenic temperature sensor (Cernox) was located near the sample in the LT-STM unit. The STM unit was cooled with the latent heat of evaporated liquid He in a pot, where liquid He was fed from a main bath via a flow valve. The temperature was changed by tuning the valve with variation rates of 4 mK/s or less. A pure H_2_ gas (≥99.999%, Westfalen AG) was dosed via a leak valve into the STM unit at a constant temperature until the intensity of the H_2_-derived peaks was saturated.^42^ For the STM tip, we used an electrochemically etched Ag wire that was further sharpened by focused-ion-beam milling.^40^ A continuous-wave 532 nm laser (*p*-polarized, 5.9 mW, Cobolt) was incident in the STM junction through a beam splitter and a fused silica window of the chamber. The laser is focused at the tip apex by adjusting the position and angle of a Ag-coated parabolic mirror mounted on a piezoelectric-motor stage in the STM unit. The parabolic mirror was also used to collect the scattered light. For Raman detection, the scattered light is directed to a spectrometer (303 mm focal length, 600 lines/mm grating, AndorShamrock 303i) outside the chamber through the fused silica window, the beam splitter, and a long-pass filter. The TER spectra shown in the main text were obtained with an accumulation time of 10 s per spectrum under the tip-height control by STM in the constant current mode with tunneling current of 1.0 nA and a sample bias voltage of 10 mV. Parameters of spectra shown in SI are described in each figure caption.

The STM experiments for the direct observation of the H_2_ 2DS were conducted in another UHV chamber (ScientaOmicron GmbH) at a constant temperature of 5 K with an electrochemically etched gold (Au) tip. Another single-crystal Ag(111) (MaTeck) was used with the same cleaning procedure as described above. The STM stage was exposed to pure H_2_ gas by opening a radiation shield, resulting in a tentative increment in the stage temperature up to ∼12 K. The STM image shown in the main text was acquired in the constant current mode with a tunneling current of 0.1 nA and a sample bias voltage of 1 V. Parameters of images shown in SI are described in each figure caption.

## Supplementary Material



## Notes

STM imaging, evaluation of laser-illumination effects, details of the island growth model, and thermodynamic and kinetic analysis based on a model including the STM-junction trapping (PDF)
