# Supplementary material for: Thermodynamics and Kinetics of Two-Dimensional H2 Gas on Ag(111) Studied by Tip-Enhanced Raman Spectroscopy
Source: Nano Lett. 2026 May 18;26(21):6868–73. doi: 10.1021/acs.nanolett.6c00325 (PMC13237815; doi:10.1021/acs.nanolett.6c00325)
Supplement: Supplementary file 1 [file nl6c00325_si_001.pdf]

Supporting Information: Thermodynamics and Kinetics of Two-Dimensional H_2 Gas on Ag(111) Studied by Tip-Enhanced Raman Spectroscopy

Shuyi Liu,[†,‡] Youngwook Park,[†] Jun Yoshinobu,[¶] Takashi Kumagai,[§,‖] Martin Wolf,[†] and Akitoshi Shiotari[*,†]

†*Department of Physical Chemistry, Fritz-Haber Institute of the Max-Planck Society, Faradayweg 4-6, 14195 Berlin, Germany*

‡*Wuhan National Laboratory for Optoelectronics, Huazhong University of Science and Technology, Wuhan 430074, China*

¶*The Institute for Solid State Physics, The University of Tokyo, 5-1-5 Kashiwanoha, Kashiwa, Chiba 277-8581, Japan*

§*Institute for Molecular Science, National Institutes of Natural Sciences, Okazaki 444-8585, Japan*

‖*The Graduate University for Advanced Studies, SOKENDAI, Hayama 240-0193, Japan*

E-mail: shiotari@fhi-berlin.mpg.de

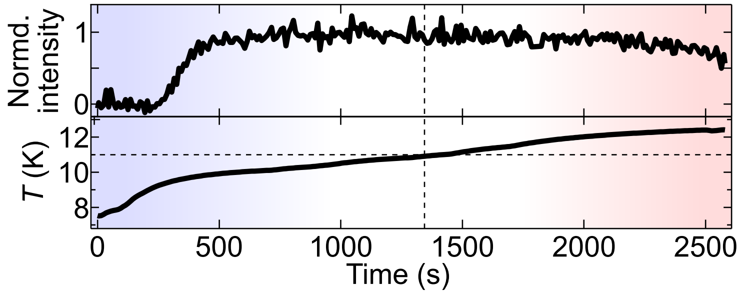

Figure S1: Time evolutions of the normalized TERS peak intensity of the H_2 rotational transition (upper panel) and the sample temperature T (bottom panel) during a monotonic heating (sample bias: 10 mV, tunneling current: 1 nA, accumulation time: 10 s per spectrum). The dotted lines represent the point when T reaches 11.0 K. The spectra at 10.2, 11.0, and 12.0 K shown in Figure 1c of the main text are recorded during this measurement.

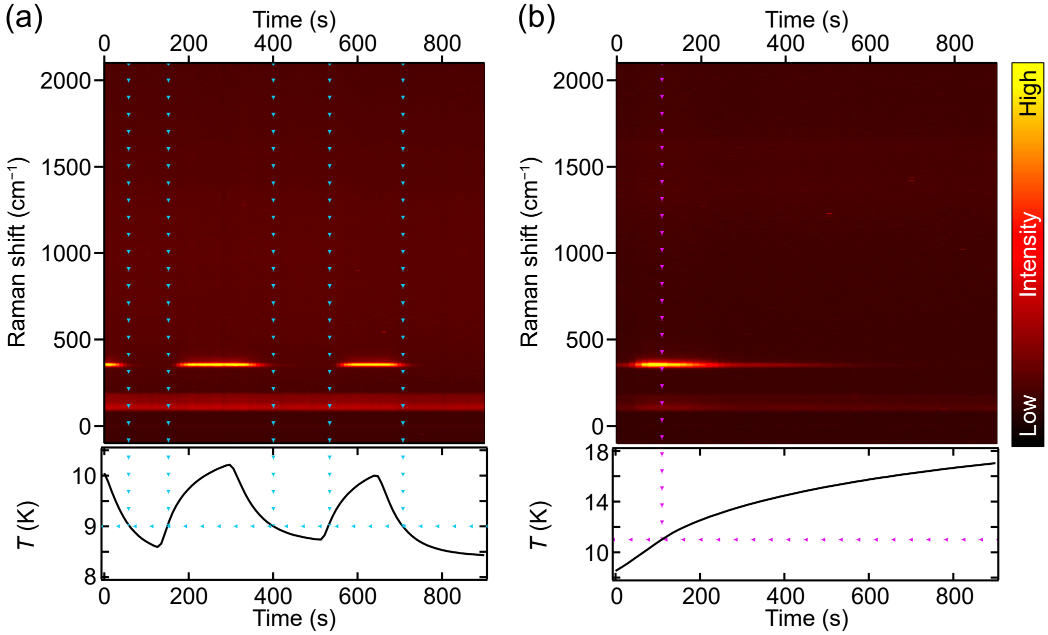

Figure S2: (a) Waterfall plot of time-dependent TERS (upper panel) and time trace of T (bottom panel) in the low-T range (sample bias: 10 mV, tunneling current: 1 nA, accumulation time: 9 s per spectrum). The measurement was conducted using a different H_2/Ag(111) sample with a different Ag tip from those in the main text. (b) Waterfall plot of TERS (upper panel) and time trace of T (bottom panel) in the high-T range (sample bias: 20 mV, tunneling current: 2 nA, accumulation time: 15 s per spectrum). The measurement was conducted using a different H_2/Ag(111) sample with a different Ag tip from those in (a) and the main text. The dotted lines represent the point when the temperature reaches 9.0 and 11.0 K for (a) and (b), respectively. Note that the constant peak at $\sim$150 cm^{-1} originates from Ag-tip phonon modes (see the main text).

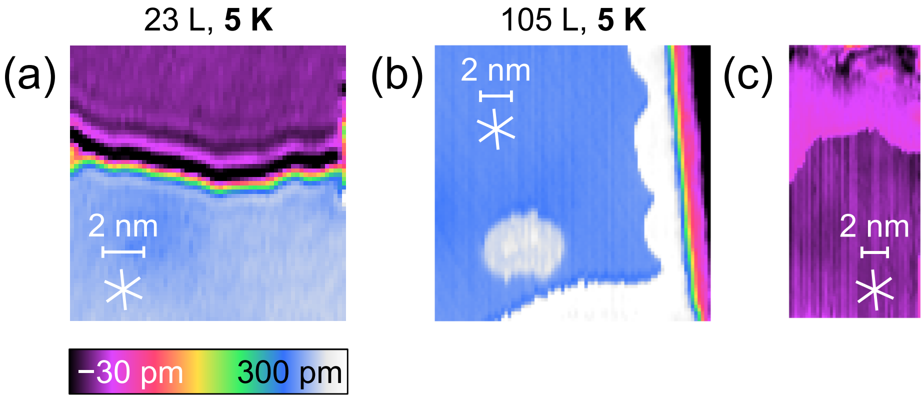

Figure S3: STM images acquired at 5 K using the STM apparatus different from the LT-TERS apparatus. (a) Ag(111) after an exposure to H_2 gas of 23 L (1 Langmuir = 10^{-6} Torr·s) (sample bias: 1 V, tunneling current: 0.1 nA). The asterisk mark indicates the $\langle 1\bar{1}0 \rangle$ directions of Ag(111). (b,c) The same sample as in (a) but after an additional exposure to H_2, 105 L in total (1 V for (b) and 0.1 V for (c), 0.1 nA). The same color bar is used for the images.

STM imaging

Figure S3 shows STM images obtained using the STM apparatus different from the LT-TERS system. At a low coverage (Figure S3a), Friedel oscillations of electrons on Ag(111) were imaged near an single atomic step, but no molecule-derived structures were observed. At a high coverage, H_2 2DS assemblies are imaged as circular islands and flat bands, as shown in Figure 2e of the main text. Figures S3b and c show images of the same sample as taht in Figure 2e, but acquired in different locations. These images indicate that the flat bands are attached at both lower and higher terraces near Ag step edges. The absence of the 2DS assemblies in the low-coverage image (Figures S3b) implies that smaller 2DS assemblies may more easily diffuse during the tip scans. This is one possible reason that the assemblies were not imaged by the LT-TERS apparatus.

For comparison, STM images acquired using the LT-TERS apparatus are shown in Figures S4 and S5. With the apparatus, neither circular islands on terraces nor flat bands near Ag step edges were observed at any exposures (0–800 L) and any temperatures (8–11 K). We note that the exposure values are not compatible between the two apparatuses because

of the different chamber layouts. Nevertheless, by TERS measurements at 10–11 K, we verified the existence of H_2 adsorbates on Ag(111) after an exposure to H_2 gas more than 30 L for the apparatus, as described in the main text. The topographic height noises of the LT-TERS apparatus are approximately 20–30 pm, which is close to a topographic height of theH_2 2DS band, 30 pm, obtained by the STM apparatus with the same setpoint parameters (Figure S3c). Therefore, we conclude that the LT-TERS apparatus cannot image the H_2 2DS assemblies.

Notably, Figures S4c and S5b show larger noises than those of the clean surface (Figures S4a and Figures S5a), which may originate from diffusing or collapsing 2DS assemblies during the scans. However, an additional exposure did not clearly enhance the noise in the image (Figures S5c), but we rather observed changes in the STM noise level by adjustments of tip-apex structures. Therefore, we cannot rule out the large contribution of the structural instability of the tip apex to the noise.

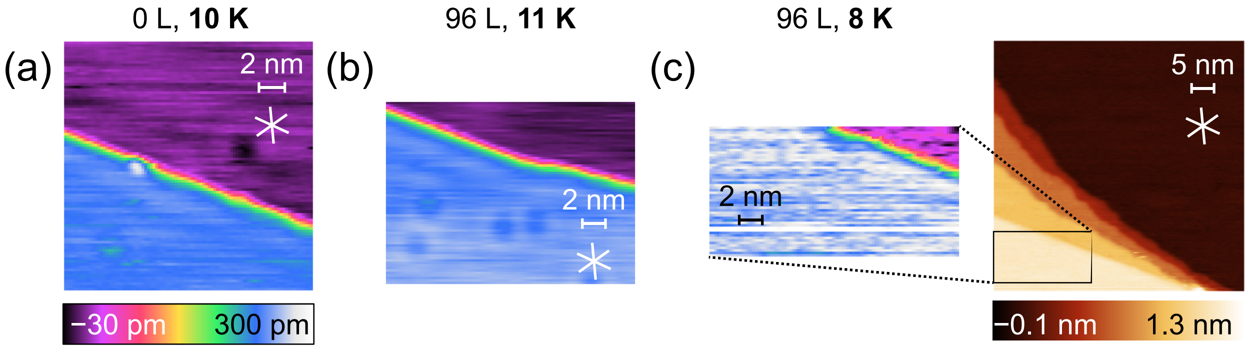

Figure S4: STM images acquired at 8–11 K by the LT-TERS apparatus. (a) Clean Ag(111) surface at 10 K (sample bias: 0.1 V, tunneling current: 0.1 nA, without laser illumination). The image is the same as that published in a previous report (Figure S1a in Supplemental Material of Ref. 1) but cropped and colorized differently. (b) The same sample as in (a) but after an exposure to H_2 gas of 96 L (0.1 V, 0.1 nA). (c) The same sample as in (b) but after a sample cooling to 8 K (0.1 V, 0.1 nA). The left panel corresponds to the magnified image in the black frame in the right panel. The same color bar is used for the images, except for the right panel of (c).

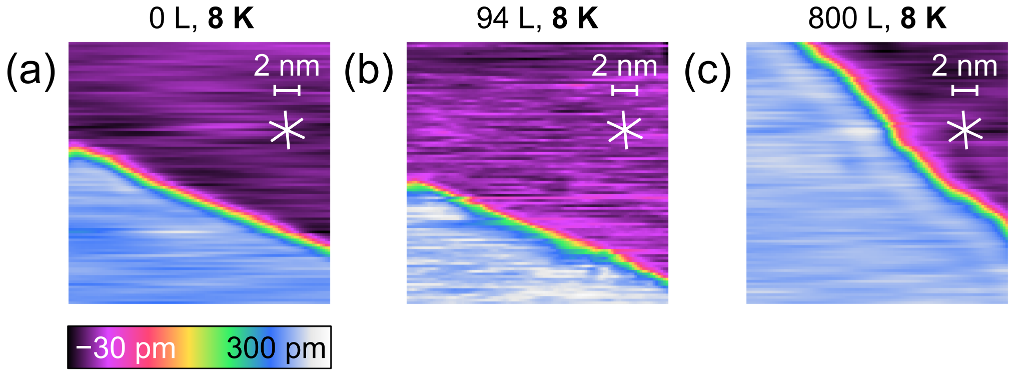

Figure S5: STM images acquired at 8 K by the LT-TERS apparatus. (a) The same sample as in Figure S4c, but after a sample heating above 20 K inducing molecular desorption (sample temperature for imaging: 8 K, sample bias: 1 V, tunneling current: 0.1 nA, without laser illumination). (b,c) The same sample as in (a) but exposures to H_2 of 94 L and ∼800 L, respectively (0.1 mV, 0.1 nA). The same color bar is used for the images.

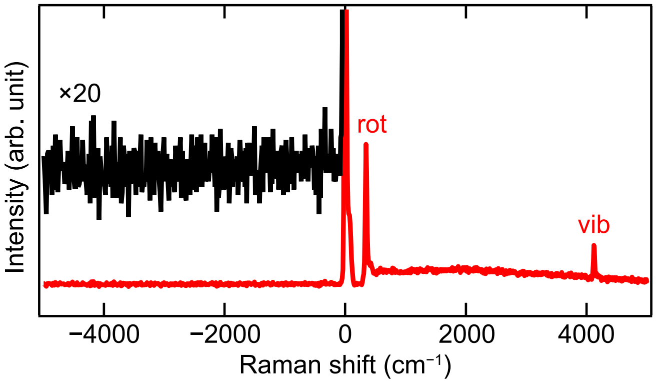

Figure S6: TER spectrum of H_2/Ag(111) in Stokes and anti-Stokes regions (sample temperature: 11.1 K, sample bias: 10 mV, tunneling current: 1 nA). The labels “rot” and “vib” indicate the Stokes Raman scattering resulting from the rotational and vibrational transitions, respectively, of H_2 on the surface.[1] The black curve shows the magnified plot in the anti-Stokes region (vertical offset for clarity).

Evaluation of laser-illumination effects

Local heating in laser-illuminated STM junctions can occur due to the decay of localized surface plasmons. The local heating has been be characterized by anti-Stokes scattering spectroscopy based on TERS or surface-enhanced Raman spectroscopy (SERS), where an anti-Stokes continuum (a broad feature in the anti-Stokes region) due to electronic inelastic light scattering[2,3] and/or anti-Stokes Raman peaks of sample materials[2,4] are detectable depending on the junction temperature.

To verify the absence of local heating, we obtained a TER spectrum of H_2/Ag(111) at 11 K including the anti-Stokes region, as shown in Figure S6. Instead of a long-pass filter, we used a notch filter to detect scattering light in both Stokes (positive wavenumbers) and anti-Stokes (negative wavenumbers) regions. In the spectrum, the Stokes region have two sharp peaks assigned to H_2 rotational and vibrational modes[1] (labeled as “rot” and “vib,” respectively). Note that due to the filter difference, the peak intensity at 0 cm^{-1} (Rayleigh scattering) is stronger and the intensity cutoff at the low wavenumber region is wider than those with the long-pass filter. The “rot” peak appears asymmetric due to the cutoff. In the anti-Stokes region (black curve), no signal exceeding the noise level was observed. This

strongly suggests that local heating effect is negligible, unlike a previous TERS study on an Ag–vacuum–Ag junction which showed an anti-Stokes continuum.[3] The previous study used tunneling currents of tens of nanoampere, much larger than that we used (1 nA). The large currents caused very narrow tip–sample junction gaps, which require the stronger near-field causing the observable local heating.

Detail of the island growth model

Here we outline the derivation of eqs 1 and 4 in the main text. Assuming that the 2DS molecules form round disk-shaped islands with a radius of r,[5,6] the number density of the 2DS molecules on the surface (N_{2DS}) is given by

$$N_{\mathrm{2DS}} = \pi \left(\frac{r}{a}\right)^2 \rho\theta_{\mathrm{2DS}}, \tag{S1}$$

where ρ is the number density of islands over the surface (*i.e.*, the number of the disk-shaped islands per unit area), θ_{2DS} is the local coverage of molecules inside the disk (*i.e.*, the number of molecules forming a 2DS island per adsorption site within the island), a is the distance of neighboring adsorption sites. Unlike atom diffusion in crystal growth,[5,6] we assume that no second layer of H_2 exists on the islands because multilayer H_2 is not stable at the temperatures.[1] The islands shrink when molecules located at the island edges (*i.e.*, 'edge molecules' located on Site 1) diffuse to empty sites outside the island (see Site -1 in Figure 2d of the main text). Since Site 1 corresponds to the circumference of the round islands, the number density of the edge molecules (N_1) is given by

$$N_1 \;=\; 2\pi \left(\frac{r}{a}\right) \rho\theta_{\mathrm{2DS}} = 2\sqrt{\pi\rho\theta_{\mathrm{2DS}} N_{\mathrm{2DS}}}. \tag{S2}$$

Conversely, the island grow when diffusing molecules in the 2DG are trapped on empty adsorption sites neighboring edge molecules by intermolecular interactions. When $\theta_{\mathrm{2DS}} < 1$,

some of Sites 1 are occupied by molecules while the others are empty. The corresponding trapping sites are thus Sites 1 without molecules and Sites -1 that are adjacent to Sites 1 with molecules. The number density, N_{-1}, is approximated by the number density of Site 1 as follows:

$$\begin{aligned} N_{-1} &= 2\pi\left(\frac{r}{a}\right)\rho(1-\theta_{\mathrm{2DS}}) + 2\pi\left(\frac{r+a}{a}\right)\rho\theta_{\mathrm{2DS}} \\ &\approx 2\pi\left(\frac{r}{a}\right) = 2\sqrt{\frac{\pi\rho N_{\mathrm{2DS}}}{\theta_{\mathrm{2DS}}}}. \end{aligned} \tag{S3}$$

Therefore,

$$\sigma_1 \equiv \frac{N_1}{\sqrt{N_{\mathrm{2DS}}}} = 2\sqrt{\pi\rho\theta_{\mathrm{2DS}}}, \tag{S4}$$

$$\sigma_{-1} \equiv \frac{N_{-1}}{\sqrt{N_{\mathrm{2DS}}}} \approx 2\sqrt{\pi\rho/\theta_{\mathrm{2DS}}}, \tag{S5}$$

$$C_1 \equiv \ln\frac{\sigma_1}{\sigma_{-1}} \approx \ln\theta_{\mathrm{2DS}}, \tag{S6}$$

where $\sigma_{\pm1}$ denote the coefficients of $\sqrt{N_{\mathrm{2DS}}}$ in $N_{\pm1}$ and C_1 denotes the constant described in the main text (eq 4). While N_{2DS} and $N_{\pm1}$ depend on T with a change in island size, the coefficients $\sigma_{\pm1}$ are independent of T. Note that eq S6 would also hold even when we assume regular hexagonal islands[7] instead of the round disks;[5,6] using the length of a hexagon's side s, N_{2DS} is given by $3(s/a)^2\rho\theta_{\mathrm{2DS}}$, leading to $\sigma_1 = 2\sqrt{3\rho\theta_{\mathrm{2DS}}}$ and $\sigma_{-1} \approx 2\sqrt{3\rho/\theta_{\mathrm{2DS}}}$.

In the low-T range, the adsorption of additional H_2 molecules from the gas phase and the desorption of molecules from the surface are negligible, *i.e.*, $N_{\mathrm{2DS}} + N_{\mathrm{2DG}} = \theta_{\mathrm{tot}}/a^2 = \text{const.}$, where N_{2DG} denotes the number density of 2DG molecules (*i.e.*, the number of diffusing molecules per unit area) and θ_{tot} is the coverage of H_2 molecules on the surface. Under the conditions, the rate equation for the equilibrium between the island growth and shrinkage is

described as

$$\begin{aligned}\frac{\mathrm{d}N_{\mathrm{2DS}}}{\mathrm{d}t} &= -k_1(1-\theta_{\mathrm{2DG}})N_1 + k_{-1}\theta_{\mathrm{2DG}}N_{-1} \\ &= -\frac{\mathrm{d}N_{\mathrm{2DG}}}{\mathrm{d}t} \\ &= 0. \end{aligned} \tag{S7}$$

As defined in the main text, θ_{2DG} is the concentration of the 2DG existing outside the disk-shaped islands, which can be expressed as

$$\theta_{\mathrm{2DG}} = \frac{\theta_{\mathrm{tot}} - \pi r^2 \rho \theta_{\mathrm{2DS}}}{1 - \pi r^2 \rho} = \frac{N_{\mathrm{2DG}} a^2}{1 - (N_{\mathrm{2DS}} a^2 / \theta_{\mathrm{2DS}})}. \tag{S8}$$

Using the Eyring–Polanyi equation, the rate constants are expressed as

$$k_i = \frac{\kappa_i k_B T}{h} \exp\!\left(\frac{\Delta S_i^*}{k_{\mathrm{B}}}\right) \exp\!\left(-\frac{\Delta H_i^*}{k_{\mathrm{B}} T}\right), \tag{S9}$$

where ΔS_i^* and ΔH_i^* are the activation entropy and enthalpy, respectively, and κ_i is the transmission coefficient for the forward ($i = 1$) and backward ($i = -1$) reactions. As shown in Figure 3f of the main text, the activation barriers are described as $\Delta H_1^* = E_{\mathrm{diff}} + \Delta H_{\mathrm{sub}}^{\mathrm{2D}}$ and $\Delta H_{-1}^* = E_{\mathrm{diff}}$. From eq S9, the equilibrium constant can be expressed as the Van't Hoff equation of

$$K_1 = \frac{\kappa_1}{\kappa_{-1}} \exp\!\left(\frac{\Delta S_{\mathrm{sub}}^{\mathrm{2D}}}{k_{\mathrm{B}}}\right) \exp\!\left(-\frac{\Delta H_{\mathrm{sub}}^{\mathrm{2D}}}{k_{\mathrm{B}} T}\right), \tag{S10}$$

where $\Delta H_{\mathrm{sub}}^{\mathrm{2D}} = \Delta H_1^* - \Delta H_{-1}^*$ is the enthalpy of the 2D sublimation as defined in the main text and $\Delta S_{\mathrm{sub}}^{\mathrm{2D}} = \Delta S_1^* - \Delta S_{-1}^*$ is the entropy of the 2D sublimation, i.e., the entropy of a 2DG molecule relative to the entropy of a 'edge molecule.' Using the constants C_1 (see eq S6) and $C_0 \equiv \Delta S_{\mathrm{sub}}^{\mathrm{2D}}/k_{\mathrm{B}} + \ln(\kappa_1/\kappa_{-1}) + C_1$, we derive eq 4 in the main text.

The line fitting of the Van't Hoff plot (Fig. 2b in the main text) provides $\Delta H_{\mathrm{sub}}^{\mathrm{2D}} = 22 \pm 1$

meV, whereas the determination of $\Delta S_{\rm sub}^{\rm 2D}$ requires the values of C_1 and κ_1/κ_{-1}. Previous theoretical calculations[1] show that a high-density adsorption structure with a coverage of ∼0.69 monolayers in the unit cell stabilizes the system. Assuming $\theta_{\rm 2DS} = 0.69$ and $\kappa_1 = \kappa_{-1}$, from eq S6, the Van't Hoff plot results in $\Delta S_{\rm sub}^{\rm 2D} \approx 2.3$ meV/K. However, this value is probably overestimated, because a previous theoretical study estimates the standard entropy of the transition from a completely trapped H_2 molecule to a freely diffusing 2DG molecule to be ∼0.8 meV/K.[8] The difference can be caused by (i) no consideration of island fragmentation and combination of the islands[5,6] and/or (ii) no consideration of molecular diffusion to the metastable adsorption sites (hcp-hollow sites[9]), which increases the possible diffusion pathways. Molecular dynamics simulations would help to better understand the diffusion and trapping process at the 2DS islands.

Reaction scheme including STM-junction trapping

Overview of the STM-junction trapping model and its results

In the main text, we treat the STM junction site equivalent to a surface adsorption site for diffusing H_2 molecules. However, it is known that a STM junction can work as a trapping site for a physisorbed molecule due to the van der Waals interaction with a metal tip.[10–12] To evaluate the tip effect, in this section, we also take into account the trapping of a diffusing molecule in the STM junction for the thermodynamic and kinetic analysis.

Based on the junction-trapping model, we analyze the energy profile of the desorption, phase transition, and junction trapping for H_2 molecules on Ag(111), as shown in Figure S7. In addition to H_2 molecules in the gas phase [$\mathbf{H_2(gas)}$], in the 2DG phase [$\mathbf{H_2(2DG)}$], and in the 2DS phase [$\mathbf{H_2(2DS)}$], we consider the junction-trapped molecule [$\mathbf{H_2(jct)}$]. Before discussing the detailed equations and analysis based on the extended model (see next subsection), we first show the conclusion; even with this more complex model than in the main text, a very similar energy diagram is obtained (Figure S7b; compare to Figure 3b of

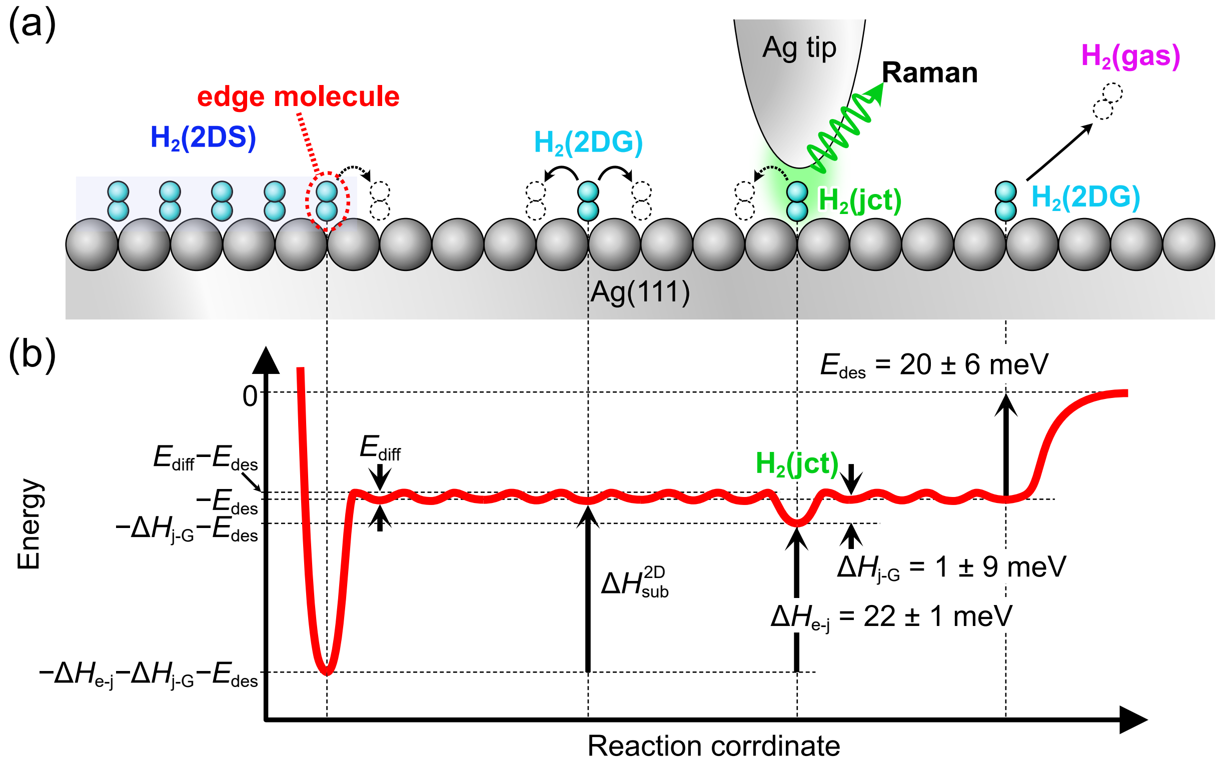

Figure S7: (a) Side-view schematic and (b) reaction coordinate diagram for an 'edge molecule' of a 2DS island to diffuse over the Ag(111) surface (**H_2(2DG)**), and to be trapped by the STM junction (**H_2(jct)**), and to desorb from the surface (**H_2(gas)**). The energy of **H_2(gas)** is set as the origin. The values displayed in the diagram represent the energies estimated in the junction-trapping model, instead of the model shown in the main text.

the main text).

In the main text, we obtained the enthalpy of the 2D sublimation ($\Delta H^{2D}_{sub} = 22 \pm 1$ meV) and the diffusion energy for the 2DG ($E_{des} = 24 \pm 2$ meV). Instead, the analysis of the TERS measurements using the junction-trapping model provides $\Delta H_{e-j} = 22 \pm 1$ meV, $\Delta H_{j-G} = 1 \pm 9$ meV, and $E_{des} = 20 \pm 6$ meV (Figure S7b), where ΔH_{e-j} is the enthalpy of transition **H_2(edg)** $\rightarrow$ **H_2(jct)**, ΔH_{j-G} is the enthalpy of transition **H_2(jct)** $\rightarrow$ **H_2(2DG)**, and E_{des} is the desorption energy from the 2DG, *i.e.*, activation barrier for **H_2(2DG)** $\rightarrow$ **H_2(gas)**. Importantly, the obtained value of $\Delta H_{j-G} \approx 1$ meV is much smaller than ΔH_{e-j} and E_{des}, but rather comparable to $E_{diff} \lesssim 1$ meV.[9] Therefore, we conclude that the effect of the Ag tip on diffusing molecules is negligibly faint, validating the simpler model used in

the main text.

The complexity of the junction-trapping model causes relatively larger errors in $\Delta H_{\mathrm{j-G}}$ and E_{des}. Nevertheless, $\Delta H_{\mathrm{j-G}} < 10$ meV is in good agreement with a previous theoretical study,[1] where an H_2 molecule in a tunneling junction between a Ag tip and Ag(111) was calculated to be stabilized by ∼4 meV compared to an isolated molecule on the surface. We note that voltage-bias application (even with a low value of 10 mV) to the junction for STM feedback and visible-laser illumination at the tip apex for the TERS measurements can also modify the potential of the junction due to the additional electric fields. Nevertheless, as reported previously,[1] the Ag–H_2 interaction is faint even when light and voltage bias are not taken into account, complicating the estimation of their contributions. A limited number of the data points for the function fitting due to the measurement difficulty is possibly responsible for the large errors; a series of $I(t, T)$ over long recording times without any tip change is required for the plot (Figures S8d and S8e). Solving the complicated rate equations of the reversible consecutive reaction[13,14] or conducting high temporal resolution TERS measurements[15] would contribute to the more accurate elucidation of the energy diagrams beyond the large fitting errors and the uncertainty originating from the simplification of the involved elementary processes.

Details of the analysis with the STM-junction trapping model

Figure S8a shows the reaction scheme in the junction-trapping model;

$$\mathbf{H_2(2DS)} \underset{k_{-1}}{\overset{k_1}{\rightleftharpoons}} \mathbf{H_2(2DG)}, \tag{S11}$$

$$\mathbf{H_2(jct)} \underset{k_{-2}}{\overset{k_2}{\rightleftharpoons}} \mathbf{H_2(2DG)}, \tag{S12}$$

$$\mathbf{H_2(2DG)} \xrightarrow{k_{\mathrm{des}}} \mathbf{H_2(gas)}, \tag{S13}$$

where $\mathbf{H_2(2DG)}$ in this model denote a diffusing molecule on an adsorption site other than the STM-junction site, $\mathbf{H_2(jct)}$ is the molecule on the junction site, and k_2 and k_{-2} denote

the rate constant of the detrapping from and trapping to the junction, respectively. In this model, only $\mathbf{H_2(jct)}$ is detectable by the local spectroscopy. The TERS peak intensity (I) is determined by the temporal occupation $\theta_{\rm jct}$ of the molecule within the single STM-junction site,

$$I = \theta_{\rm jct} I_{\rm max}. \tag{S14}$$

Notably, even when we distinguish between $\mathbf{H_2(2DG)}$ and $\mathbf{H_2(jct)}$ in the model, we still need to consider the existence of the 2DS islands to explain the observed TERS results in the low-T range; $I = 0$ below 9 K (Figure 1b of the main text) suggests the existence of a thermodynamically more stable phase than $\mathbf{H_2(jct)}$ to strongly trap diffusing H_2 molecules.

Low-T range: The 2DS–2DG transition is expected to occur between 9 and 10 K (Figures S8b), while the desorption is negligible in the low-T range (blue box in Figure S8a). In a similar manner to eq S7, the rate equations are described as follows;

$$\frac{\mathrm{d}N_{\rm 2DS}}{\mathrm{d}t} = -k_1\sigma_1(1-\theta_{\rm 2DG})\sqrt{N_{\rm 2DS}} + k_{-1}\sigma_{-1}\theta_{\rm 2DG}\sqrt{N_{\rm 2DS}} = 0, \tag{S15}$$

$$\frac{\mathrm{d}\theta_{\rm jct}}{\mathrm{d}t} = -k_2\sigma_2(1-\theta_{\rm 2DG})\theta_{\rm jct} + k_{-2}\sigma_{-2}\theta_{\rm 2DG}(1-\theta_{\rm jct}) = 0, \tag{S16}$$

$$\frac{\mathrm{d}N_{\rm 2DG}}{\mathrm{d}t} = -\left(\frac{\mathrm{d}N_{\rm 2DS}}{\mathrm{d}t} + \frac{\mathrm{d}\theta_{\rm jct}}{\mathrm{d}t}\right) = 0, \tag{S17}$$

where σ_2 and σ_{-2} are the coefficients attributed to the distributions of the possible adsorption sites for H_2 diffusing from and to the junction, respectively, and $\sigma_2 = \sigma_{-2}$ holds for the single site of the STM junction. From eqs S15 and S16,

$$K_1 \equiv \frac{k_1}{k_{-1}} = \frac{\sigma_{-1}}{\sigma_1}\frac{\theta_{\rm 2DG}}{1-\theta_{\rm 2DG}}, \tag{S18}$$

$$K_2 \equiv \frac{k_2}{k_{-2}} = \frac{\theta_{\rm 2DG}}{1-\theta_{\rm 2DG}}\frac{1-\theta_{\rm jct}}{\theta_{\rm jct}}. \tag{S19}$$

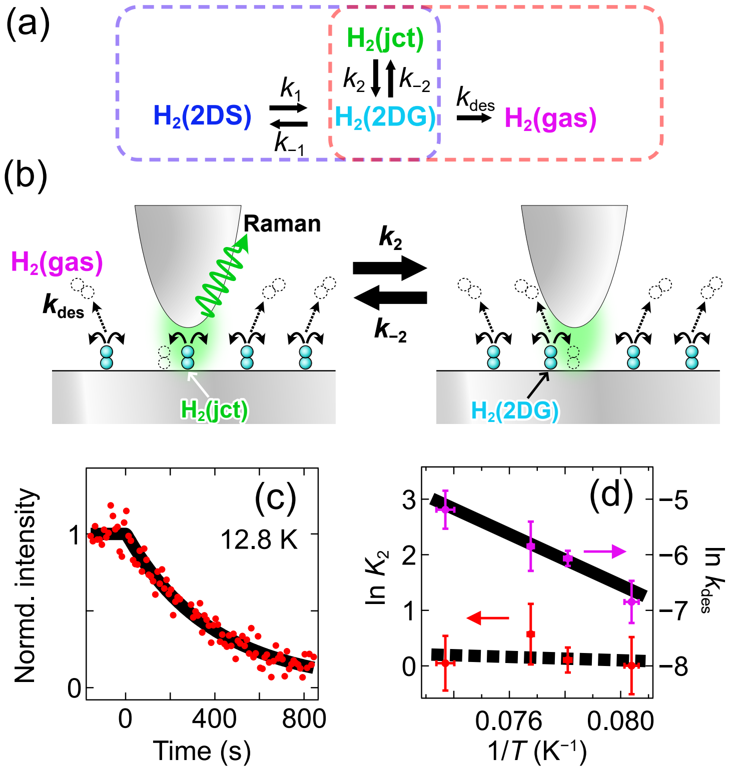

Figure S8: (a) Reaction scheme with the consideration of $\mathbf{H_2(jct)}$. (b) Schematic of the steady state for $\mathbf{H_2(2DG)}$ and $\mathbf{H_2(jct)}$ during the desorption process in the high-T range (red dotted box in (a)). (c) The same $I(t)$ curve as that in Figure 3c of the main text, but with the fitting curve with a different function, eq S24 (black curve). (d) Van't Hoff plot for the $\mathbf{H_2(jct)}$–$\mathbf{H_2(2DG)}$ transition (red bullets with the dotted fitting line, left axis) and Arrhenius plot for the desorption (magenta bullets with the solid fitting line, right axis) based on the scheme in (b). The fitting parameters are $\Delta H_{\mathrm{j-G}} = 1.3 \pm 8.6$ meV and $E_{\mathrm{des}} = 19.7 \pm 6.2$ meV.

We can remove $\theta_{\rm 2DG}$ from the equation by considering the equilibrium constant $K_1' \equiv K_1/K_2$. Using eqs S14, S18, and S19, we obtain

$$K_1'(T) = \frac{\sigma_{-1}}{\sigma_1}\frac{I(T)}{I_{\rm max} - I(T)}. \tag{S20}$$

The rate constants $k_{\pm 2}$ are expressed by eq S9 with the transmission coefficients $\kappa_{\pm 2}$ and activation entropy $\Delta S^*_{\pm 2}$ and enthalpy $\Delta H^*_{\pm 2}$. As shown in Figure S7b, $\Delta H^*_2 = E_{\rm diff} + \Delta H_{\rm j-G}$ and $\Delta H^*_{-2} = E_{\rm diff}$. From eq S9, the equilibrium constant K_1' is expressed as

$$K_1' = \frac{\kappa_1\kappa_{-2}}{\kappa_{-1}\kappa_2}\exp\left(\frac{\Delta S_{\rm e-j}}{k_{\rm B}}\right)\exp\left(-\frac{\Delta H_{\rm e-j}}{k_{\rm B}T}\right), \tag{S21}$$

where $\Delta S_{\rm e-j} \equiv \Delta S^*_1 - \Delta S^*_2$ and $\Delta H_{\rm e-j} \equiv \Delta H^*_1 - \Delta H^*_2$ are the entropy and enthalpy of transition $\mathbf{H_2(edg)} \rightarrow \mathbf{H_2(jct)}$, respectively (see Figure S7b). From eqs S20 and S21, we obtain

$$\begin{aligned} \ln\frac{I(T)}{I_{\rm max} - I(T)} &= \ln K_1'(T) + C_1 \\ &= -\frac{\Delta H_{\rm e-j}}{k_{\rm B}}\cdot\frac{1}{T} + C_0', \end{aligned} \tag{S22}$$

where $C_0' \equiv \Delta S_{\rm e-j}/k_{\rm B} + \ln(\kappa_1/\kappa_{-1}) - \ln(\kappa_2/\kappa_{-2}) + C_1$ is a constant independent of T. Equation S22 is identical to eq 4 of the main text except for K_1' versus K_1, $\Delta H_{\rm e-j}$ versus $\Delta H^{\rm 2D}_{\rm sub}$, and C_0' versus C_0. Therefore, the Van't Hoff plot shown in Figure 2b for eq 4 of the main text can be used for eq S22 in the same manner, and the fitting results in $\Delta H_{\rm e-j} = 22 \pm 1$ meV.

High-T range: Above 12 K, the 2D sublimation (2DS-to-2DG transition) is accelerated, as described in the main text. In the high-T range, we thus consider only the reversible transition between $\mathbf{H_2(2DG)}$ and $\mathbf{H_2(jct)}$ and the irreversible desorption of $\mathbf{H_2(2DG)}$ (red box in Figure S8a). At 13–14 K, the desorption undergoes for several minutes, indicating

that the desorption is the rate-determining step with a sufficiently small k_{des}. Therefore, the conditions observed in each spectrum can be regarded as a steady state for the intermediate, *i.e.*, $\mathbf{H_2(2DG)}$, in the sequential reaction of eq S12 followed by eq S13. In other words, the peak decay results from both the slow change in the 2DG concentration and the approximate equilibrium condition between $\mathbf{H_2(2DG)}$ and $\mathbf{H_2(jct)}$ within the accumulation time per spectrum (Figure S8c).

Under the first-order desorption, the 2DG concentration θ_{2DG} can be expressed as $\theta_{\mathrm{2DG}}(t,T) = \exp[-k_{\mathrm{des}}(T)t]$, where $t = 0$ indicates the time when gas exposure was stopped. The first-order desorption has been adopted in the models of crystal growth and thin-film formation,[5–7] and is also supported by a previous study on temperature programmed desorption of H_2 from Ag(111).[16] Assuming that the desorption is slow and negligible in a short time window as described above, from eqs S14 and S19, the equilibrium constant K_2 can be expressed as

$$K_2(T) = \frac{\theta_{\mathrm{2DG}}(t,T)}{1-\theta_{\mathrm{2DG}}(t,T)} \frac{I_{\mathrm{max}} - I(T)}{I(T)} \, . \tag{S23}$$

From eq S23, therefore, the peak intensity is

$$I(t,T) = \frac{I_{\mathrm{max}}}{K_2(T)\{\exp[k_{\mathrm{des}}(T)t] - 1\} + 1} \, . \tag{S24}$$

K_2 and k_{des} are responsible for the steepness of the intensity drop at $t \approx 0$ and the decay rate, respectively, in an $I(t)$ curve at a constant T. The solid curve in Figure S8d shows the same $I(t)$ curve as that in Figure 2c of the main text but with the fitting result using eq S24 (black curve). Fitting the $I(t)$ curves at several constant temperatures with eq S24 provide the series of $k_{\mathrm{des}}(T)$ and $K_2(T)$, allowing for a Van't Hoff plot $K_2(T) = (\kappa_2/\kappa_{-2}) \cdot \exp(\Delta S_{\mathrm{j-G}}/k_{\mathrm{B}}) \cdot \exp(-\Delta H_{\mathrm{j-G}}/k_{\mathrm{B}}T)$ and an Arrhenius plot $k_{\mathrm{des}}(T) = A\exp(-E_{\mathrm{des}}/k_B T)$. From the two plots (Figure S8e), we obtain $\Delta H_{\mathrm{j-G}} = 1 \pm 9$ meV and $E_{\mathrm{des}} = 20 \pm 6$ meV (see Figure S7b).

References

(1) Shiotari, A.; Liu, S.; Trenins, G.; Sugimoto, T.; Wolf, M.; Rossi, M.; Kumagai, T. Picocavity-Enhanced Raman Spectroscopy of Physisorbed H_2 and D_2 Molecules. *Phys. Rev. Lett.* **2025**, *134*, 206901.

(2) Hugall, J. T.; Baumberg, J. J. Demonstrating photoluminescence from Au is electronic inelastic light scattering of a plasmonic metal: the origin of SERS backgrounds. *Nano Lett.* **2015**, *15*, 2600–2604.

(3) Liu, S.; Hammud, A.; Wolf, M.; Kumagai, T. Anti-stokes light scattering mediated by electron transfer across a biased plasmonic nanojunction. *ACS Photon.* **2021**, *8*, 2610–2617.

(4) Liu, S.; Wolf, M.; Kumagai, T. Nanoscale heating of an ultrathin oxide film studied by tip-enhanced Raman spectroscopy. *Phys. Rev. Lett.* **2022**, *128*, 206803.

(5) Le Lay, G.; Kern, R. Physical methods used for the characterization of modes of epitaxial growth from the vapor phase. *J. Crystal Growth* **1978**, *44*, 197–222.

(6) Bondarenko, V.; Kuz'min, M.; Mittsev, M. Kinetics of desorption from adsorbed layers formed by two-dimensional islands and by a two-dimensional gas. *Phys. Solid State* **2001**, *43*, 1172–1177.

(7) Frankl, D.; Venables, J. Nucleation on substrates from the vapour phase. *Adv. Phys.* **1970**, *19*, 409–456.

(8) Savara, A. Standard states for adsorption on solid surfaces: 2D gases, surface liquids, and Langmuir adsorbates. *J. Phys. Chem. C* **2013**, *117*, 15710–15715.

(9) Kunisada, Y.; Kasai, H. Hindered rotational physisorption states of H_2 on Ag(111) surfaces. *Phys. Chem. Chem. Phys.* **2015**, *17*, 19625–19630.

(10) Wang, H.; Li, S.; He, H.; Yu, A.; Toledo, F.; Han, Z.; Ho, W.; Wu, R. Trapping and characterization of a single hydrogen molecule in a continuously tunable nanocavity. *J. Phys. Chem. Lett.* **2015**, *6*, 3453–3457.

(11) Merino, P.; Rosławska, A.; Leon, C. C.; Grewal, A.; Große, C.; González, C.; Kuhnke, K.; Kern, K. A single hydrogen molecule as an intensity chopper in an electrically driven plasmonic nanocavity. *Nano Lett.* **2018**, *19*, 235–241.

(12) Wang, L.; Xia, Y.; Ho, W. Atomic-scale quantum sensing based on the ultrafast coherence of an H_2 molecule in an STM cavity. *Science* **2022**, *376*, 401–405.

(13) Rashid, A.; Hasan, O.; Siddique, U.; Tahar, S. Formal reasoning about systems biology using theorem proving. *PLos ONE* **2017**, *12*, e0180179.

(14) Takahashi, S.; Abe, T.; Sato, H.; Hiraoka, S. Pathway bias and emergence of quasi-irreversibility in reversible reaction networks: Extension of Curtin-Hammett principle. *Chem* **2023**, *9*, 2971–2982.

(15) Luo, Y.; Martin-Jimenez, A.; Gutzler, R.; Garg, M.; Kern, K. Ultrashort pulse excited tip-enhanced Raman spectroscopy in molecules. *Nano Lett.* **2022**, *22*, 5100–5106.

(16) Sugimoto, T.; Fukutani, K. Effects of rotational-symmetry breaking on physisorption of ortho-and para-H_2 on Ag(111). *Phys. Rev. Lett.* **2014**, *112*, 146101.
